# Pregnant and Breastfeeding Mothers Who Have Received the ChAdOx1 AstraZeneca COVID-19 Vaccine May Have Infants with an Increased Risk of Zinc Insufficiency and SCID Disease

**DOI:** 10.34172/apb.2023.024

**Published:** 2022-04-04

**Authors:** Amr Ahmed, Amr Ghit, Mahmoud El Kazzaz

**Affiliations:** ^1^Department of Public Health, Tuberculosis Program, First Health Cluster, Ministry of Health, Riyadh, Saudi Arabia.; ^2^Department of Medicine and Aging Sciences, “G. d’Annunzio” University of Chieti-Pescara, Chieti, Italy.; ^3^Center for Advanced Studies and Technology (CAST), “G. d’Annunzio” University of Chieti-Pescara, Chieti, Italy.; ^4^Department of Chemistry and Biochemistry, Faculty of Science, Damietta University, Egypt.

## Dear Editor,

 Adenosine deaminase, referred to as ADA with Enzyme Commission number (EC) 3.5.4.4 (ADA, EC 3.5.4.4), is a monomeric enzyme that plays a key role in purine metabolism, catalyzes the irreversible deamination of adenosine to inosine.^1^ Additionally, it acts as a co-stimulator, a cell-to-cell connector, and an allosteric modulator.^2^ ADA is found in all human tissues, and aberrant levels are linked to a variety of human disorders. DNA synthesis is inhibited due to ADA’s relatively low activity. Moreover, low activity of ADA leads to accumulation of lymph-toxic deoxyadenosine triphosphate inducing apoptosis in immature thymocytes.^3,4^

 It is widely recognized that ADA deficiency leads to severe combined immunodeficiency disease (SCID), which is linked with severe lymphopenia and dysfunction of natural killer, B, and T cells.^5^ A high amount of ADA activity, on the other hand, can lead to a variety of severe disorders such as tuberculous peritonitis and leukemia.^6^

 Approximately 30% of proteins in cells form complexes with metal ions, which are required not only for regulating biological activity but also for protein stabilization. ADA comprises a strongly bonded zinc ion that is required for its function.^7^ Several crystal structures of ADA have been discovered, each featuring a firmly bound Zn^2+^ that is crucial for the native protein’s stability and catalytic activity.^8,9^ A previous study examines the effect of Zn^2+^ on the maintenance of ADA. The protein’s crystallographic structure preserved a high degree of order and homology to the original structure when Zn^2+^ was removed, with some modifications localized to the regions bordering the pocket. However, the protein’s stability was dramatically reduced.^7^

 EDTA is a highly potent zinc-chelating agent used in protein interaction studies. EDTA strongly binds with Zn^2+^ compared to other divalent metals such as Mg^2+^. The possibility for EDTA treatment to disrupt the structure and interactions of zinc-binding proteins must be recognized. Using EDTA to eliminate endogenic zinc seems to provide a technique for investigating the consequences of short-term zinc supplementation during crucial stages of pregnancy in zinc-deficient rats.^10^ A study demonstrated the binding capabilities of transcription factors of the zinc-binding module C/H1 (cysteine/histidine-rich region 1) discovered in p300 and the transcriptional co-activator protein CBP (CREB-binding protein). Their significant findings have shown that treating native C/H1 with EDTA results in irreversible denaturation and aggregation. Moreover, they discovered that the released form of C/H1 engages in nonspecific protein-protein interactions. Excess Zn^2+^ did not restore C/H1 to its normal structure, emphasizing the permanent denaturation caused by EDTA. EDTA’s remarkable affinity for these metals is due to a high-speed and essentially diffusion-limited association rate paired with a prolonged dissociation rate.^11^

 Of note, PEGylation of adenoviral vectors may be a viable technique in terms of safety profile because it has lowered IL-6 and liver toxicity significantly; however, it requires some optimization.^12^ Concerns have been raised about AstraZeneca’s COVID-19 vaccine’s excipients, which include polysorbate 80 (a stabilizer) and EDTA (a binding agent).^13^ Likewise, EDTA was discovered to destroy breast milk cells, damage the milk fat globule membrane, disrupt membrane-bound proteins, free fatty acids, and lower pH. Furthermore, it led to false-positive hemolytic tests. EDTA is widely used in modern pharmacy and medicine. However, few investigations have been conducted to determine the cytotoxic consequences of EDTA on biological samples.^14^ Also, it was hypothesized that EDTA bound free zinc, preventing it from being exchanged for integrated zinc. EDTA caused zinc deficiency, which has teratogenic implications. The teratogenic effect of EDTA might be attributed to chelation, which resulted in mineral reduction and mineral deficiency in the developing fetus.^11^

 The activities of thymidine kinase and DNA polymerase were reduced in rabbit hepatocytes, while ADA and dehydrogenase activities were unchanged. The diadenosine tetraphosphate concentration was lower in EDTA-treated cells. Zinc chloride inhibited hydrolase activity by increasing diadenosine tetraphosphate synthesis. The cation’s ability to reverse DNA synthesis inhibition has led to speculation that zinc is a possible second messenger for triggering mitogenicity.^15^ This finding ensures that DNA synthesis was hindered because of the impairment of replication enzymes by EDTA. Furthermore, pregnant rats that were administered 3% disodium EDTA at different pregnancy stages developed severe deformities when administered 100 ppm zinc rather than 1000 ppm zinc. There was no evidence of fetal or maternal toxicity with ZnCaEDTA (8 mmol/m^2^/day) or ZnEDTA (8 and 20 mmol/m^2^/day). EDTA was supplied via gavage and in feed to rats on GDs 7–14 at 1000 mg/kg/d and 1250 mg/kg/d, respectively, and resulted in progeny abnormalities and maternal poisoning. However, in mice, offspring of dams orally fed 1000 mg/kg/d EDTA on GDs 8–12 exhibited no abnormalities. It has been proposed that zinc inadequacy due to EDTA causes teratogenic consequences.^15^

 Briefly, zinc is essential for the activity and stability of numerous enzymes such as ADA, which may cause health concerns if deficient. EDTA is an effective zinc chelator that can impair ADA stability. Although the teratogenic effect of disodium EDTA may be due to metal chelation. Therefore, we strongly suggest that clinical trials are needed to monitor the health condition of infants born to mothers who have received the ChAdOx1 AstraZeneca COVID-19 vaccine.

## Competing Interests

 The authors declare no conflict of interest in this study.

## Ethical Approval

 Not applicable.
